# Identification and preliminary characterization of chemosensory perception-associated proteins in the melon fly *Bactrocera cucurbitae* using RNA-seq

**DOI:** 10.1038/srep19112

**Published:** 2016-01-11

**Authors:** Samia Elfekih, Chien-Yu Chen, Ju-Chun Hsu, Mahdi Belcaid, David Haymer

**Affiliations:** 1 Commonwealth Science and Industry Organization (CSIRO), Biosecurity flagship, P.O. BOX 1700, Canberra, ACT 2601, Australia; 2 National Taiwan University, Department of Bio-industrial Mechatronics and Engineering, Taipei, Taiwan; 3 National Taiwan University, Department of Entomology, Taipei, Taiwan; 4 Information and Computer Sciences, University of Hawaii at Manoa, Honolulu, Hawaii, USA; 5 Department of Cell and Molecular Biology, University of Hawaii at Manoa, Honolulu, Hawaii, USA

## Abstract

An investigation into proteins involved in chemosensory perception in the melon fly, *Bactrocera cucurbitae* (Diptera: Tephritidae) is described here using a newly generated transcriptome dataset. The melon fly is a major agricultural pest, widely distributed in the Asia-Pacific region and some parts of Africa. For this study, a transcriptome dataset was generated using RNA extracted from 4-day-old adult specimens of the melon fly. The dataset was assembled and annotated via Gene Ontology (GO) analysis. Based on this and similarity searches to data from other species, a number of protein sequences putatively involved in chemosensory reception were identified and characterized in the melon fly. This included the highly conserved “*Orco*” along with a number of other less conserved odorant binding protein sequences. In addition, several sequences representing putative ionotropic and gustatory receptors were also identified. This study provides a foundation for future functional studies of chemosensory proteins in the melon fly and for making more detailed comparisons to other species. In the long term, this will ultimately help in the development of improved tools for pest management.

Sophisticated mechanisms of chemosensory perception are essential to many aspects of insect behavior and survival. Insects must be able to detect the presence of specific chemicals in their environment over relatively long distances to find food sources and potential mates. In addition to these more universal aspects of chemosensory perception, the evolutionary trajectories followed by individual species to exploit different aspects of their environment, such as the presence of specific host plants and/or encounters with conspecific mates, are most likely reflected in the presence of defined sets of species-specific genes and proteins involved in chemosensory perception^1^,^2^.

Beginning primarily with work on *Drosophila melanogaster*, a considerable amount of information about genes and proteins involved in chemosensory perception specific to insects has been developed^3^. At the genome level in particular, the *Drosophila* studies have provided tremendously valuable reference sets of information to facilitate mining for similar datasets in a wide range of other insects^4^,^5^,^6^. Among these references, are sets of protein sequences, predicted mainly from transcriptome studies, involved in critical aspects of chemosensory perception such the odorant receptors and odorant binding proteins (OBPs), the ionotropic receptor (IR) proteins, and the taste or gustatory receptor (GR) proteins^7^.

Beyond *Drosophila*, however, only limited information is available for species such as those in the family Tephritidae that include many major agricultural pests^8^,^9^,^10^.

One of these is the melon fly, a highly polyphagous pest that attacks a wide range of economically important cucurbit crops^11^.

Here, using a newly generated transcriptome dataset, we have identified putative chemosensory proteins in this species and compared them to counterparts in congeneric species such as the oriental fruit fly, *Bactrocera dorsalis* and more distantly related species within the family Tehpritidae such the Mediterranean fruit fly *Ceratitis capitata*, the apple maggot *Rhagoletis pomonella* and the walnut husk fly *R. suavis*. These species, although also classified as economic pests^12^,^13^,^14^, are known to show both qualitative and quantitative differences in critical aspects of chemosensory perception. In addition, where appropriate, alignments to protein and gene sequences from *Drosophila*, a non-economically important species, were also made.

## Results

### Transcriptome assembly

The newly-generated melon fruit fly transcriptome was derived from RNA obtained from a total of 30 four-day old melon fly adults. The assembly was performed using Trinity, with an optimal K-mer length set to 25. The assembly generated 55,141 isotigs with a minimum sequence length of 100 bp. The N50 was 3,117 bp and the average contig length was 1,469 bp (Table 1). The transcriptome assembly was deposited in the SRA database and is available under accession identifier SRP058791.

### Comparison of *B. cucurbitae transcriptome* to published datasets

In order to assess the quality of our assembled transcriptome, we compared our results to those obtained from the combined assembly of four *B. cucurbitae* datasets, produced from egg, larvae, pupae and adult stages (SRAS: SRS691534, SRS691533 SRS691532 and SRS691531 respectively). While both our assembly and the one previously published^15^ yielded similar GC rates (~39%), our results contained 14,863 and 21,062 fewer unigenes and isoforms respectively. Approximately 21.4% of the difference in isoforms was attributed to transcripts shorter than 300 bp, and 37.7% of the difference consisted of transcripts ranging in length between 300 and 800 bp. Additionally, our assembly identified 227 more transcripts of length greater than 10,000 bp and more proteins with a complete ORFs (25,943 in our assembly versus 12,936 in the published dataset^15^. The presence of fewer short transcripts in favor of longer ones could be an indication of a less fragmented assembly, which can be supported by our higher N50 (3,117 bp), versus 2,626 bp in the assembly retrieved from Genbank^15^. Based on this comparative analysis, the present melon fly transcriptome is of high quality and can, therefore, be used to investigate aspects related to the biology of this important species, such as the chemosensory perception genes and proteins.

### Gene Ontology analysis

The Blast2GO annotation was used, and the results were visualized in the protein classification system PANTHER (http://www.pantherdb.org)^16^,^17^. A total of 25,943 transcripts were predicted, which represents 47% of the total number of generated contigs (55,141). Of these predicted transcripts, 19,071 (73%) were associated with GO terms and 4,661 transcripts were assigned to three main GO classes, specifically: Biological process (1,918), molecular function (1,086) and cellular components (98) (Supplementary material Fig. S1). Within the molecular function class, the most abundant GO associations were linked to catalytic (GO:0003824) [32.9%] and binding (GO:0005488) activities [32.1%]. At the biological process level, the metabolic process (GO: 0008152) was the most abundant association [27%] followed by cellular process (GO:0009987) [15.10%]. Other GO terms, such as hydrolase, transferase, transcription factor and nucleic acid binding proteins were also represented but at relatively lower levels of abundance within the dataset.

Further annotation of the transcriptome of the melon fruit fly permitted the extraction of transcripts corresponding to putative chemosensory genes and gene families and their corresponding proteins as described in the next sections.

### Candidate chemosensory genes

A total number of 52 sequences were extracted and were putatively classified as follows: 13 Odorant-Binding Proteins (OBPs) and 1 Odorant receptor co-receptor (Bcu*Orco*), 31 Ionotropic Glutamate Receptors (iGluRs), and 7 Gustatory receptors (GRs).

### OBP proteins

**Figure 1:**
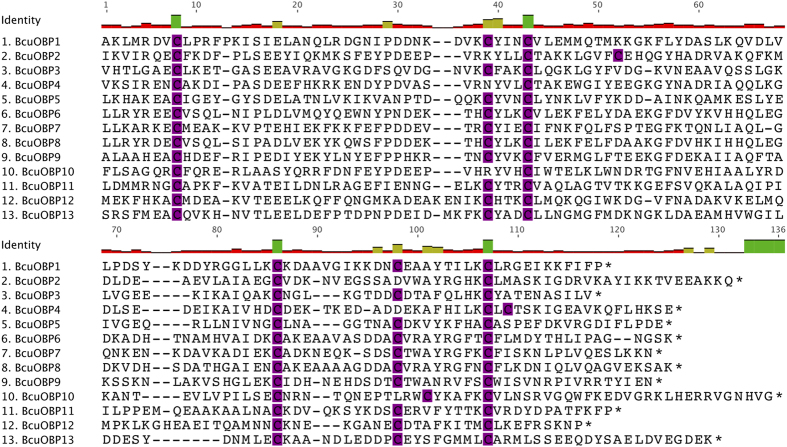
Alignment of the Odorant –Binding protein putative sequences of *B*. *cucurbitae*. The conserved cysteine motifs are highlighted in purple.

The 13 candidate OBP proteins were further characterized by initially aligning them to each other (Fig. 1) and to similar sequences from *D. melanogaster.* This allowed us to classify them using already established nomenclature^18^ and to organize them into different classes based on key features, such as the number of cysteine motifs present in each transcript.

The predicted OBPs were grouped into two main classes based on the following descriptors: First, “Classic” OBPs that contain six cysteine motifs at conserved positions. This class also includes the antennal binding proteins (ABPs). Second, the “Minus-C” class that include sequences lacking 2 cysteine residues, usually C2 and C5^18^. Overall, we detected three OBPs belonging to the Minus-C class and 10 falling into the “Classic” category. Members of an additional class designated “Plus-C” that possess 4 to 6 more cysteine residues were not identified in our dataset.

**Figure 2:**
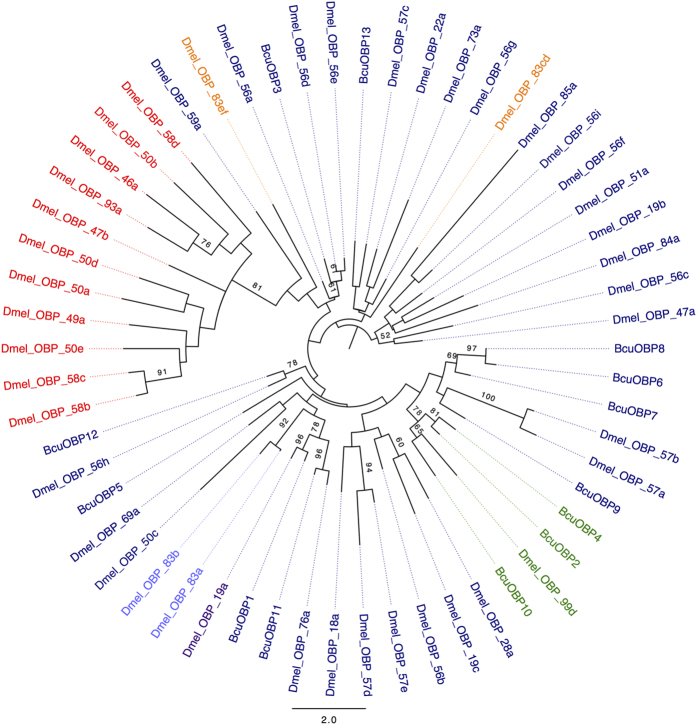
Maximum Likelihood Phylogenetic tree of *B.* *cucurbitae* and *D.* *melanogaster* OBPs. Bootstrap values greater than 50% are displayed (1000 replications). The colors refer to the OBP class (blue: Classical, red: Plus-C, green: Minus-C, orange: Dimer, Grape: ABPII).

We used all the putative OBPs from *B. cucurbitae* and representative homologous sequences from *D. melanogaster* to build a Maximum Likelihood phylogenetic tree (Fig. 2). The tree shows a clear cluster representing the Minus-C OBP class described previously consisting of three of the BcuOBPs (BcuOBP2, BcuOBP4, BcuOBP10) with specific counterparts from *D. melanogaster* (Dmel_OBP_99d). This result also shows a large clade containing the classic OBPs, and there appears to be an expansion of sequences from *B. cucurbitae* including BcuOBP6, BcuOBP7, BcuOBP8, BcuOBP9, clustering with the *Drosophila* sequence Dmel_OBP57a and Dmel_OBP57b.

The Bcu_OBP13 protein clusters with Dmel_OBP57c, while the BcuOBP1 sequence clusters with Dmel_OBP19a, which is known to be an antennal binding protein. Another of the classical OBP protein identified here (Bcu_OBP11) appears to be closely related to the sequence corresponding to Dmel_OBP76a. This is also known as LUSH, a protein involved in pheromone binding activities.

**Figure 3:**
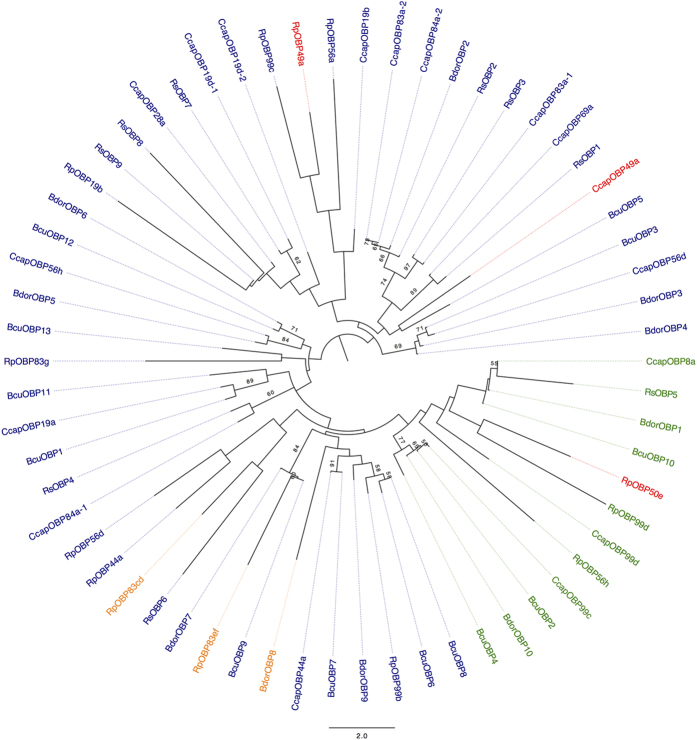
Maximum Likelihood Phylogenetic tree of Tephritid fruit flies Odorant-Binding Proteins (OBPs). Bootstrap values greater than 50% are displayed (1000 replications).

A protein BLAST analysis comparing the putative OBPs from the melon fly to their counterparts in *B. dorsalis*, a congeneric species, was also performed. Table 2 shows the results. Here, the similarity values range from 20 to 93%. In order to compare the melon fruit fly OBPs to other species from the Tephritidae family, we built a Maximum Likelihood phylogenetic tree using the 13 BcuOBPs identified and OBP sequences of other tephritid fruit flies already published and were retrieved from Genbank (Supplementary Material Table S1). These included 11 OBPs from the congeneric species *B. dorsalis,* designated as BdorOBPs^19^, 17 OBP from the Mediterranean fruit fly *C. capitata,* designated as Ccap OBPs^20^, 15 OBP from the apple maggot fly *Rhagoletis pomonella* designated as RpOBPs^21^ and 9 OBP from *R. suavis* designated as RsOBPs^22^. The mid-point rooted ML tree (Fig. 3) shows clustering by OBP class. The melon fly Minus-C OBPs (BcuOBP2, BcuOBP4) cluster with their homologues in the oriental fruit fly (BdorOBP10) and in the medfly (CcapOBP99c). Within the same Minus-C clade, another cluster harbors BcuOBP10 and CcapOBP8a. The majority of the remaining OBPs belong to the classic clade and are grouped according to their percentage of similarity among the tephritid species. The Dimer and Plus-C OBPs are scattered among the classic OBPs clade, since we did not detect any OBPs belonging to those two classes in the melon fly.

### *Orco* gene

**Figure 4:**
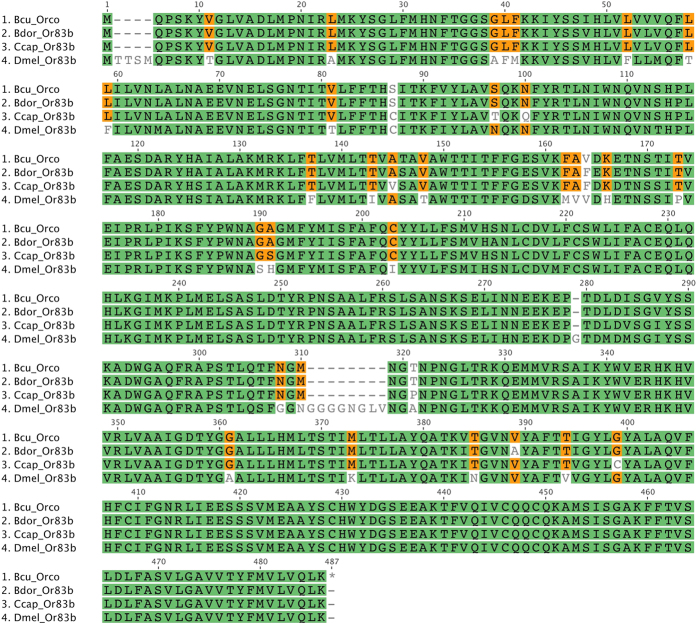
Alignment of the Odorant receptor co-receptor (*Orco*) protein sequences corresponding to *B.* *cucurbitae*, B. *dorsalis* and D. *melanogaster.*

The odorant receptor co-receptor in *B. cucurbitae* (*BcuOrco*) was identified as a protein sequence with 473 amino acids. When aligned with homologs from other fruit fly species (Fig. 4) i.e. *B. dorsalis* (ACC86853) and *C. capitata* (XP_012156143) and to *D. melanogaster* (NP_524235), the highest percentage of identity is scored between *B. cucurbitae* and *B. dorsalis*, (98%), followed by *C. capitata* (96%), then *D. melanogaster* (87%).

### Glutamate receptor proteins

**Figure 5:**
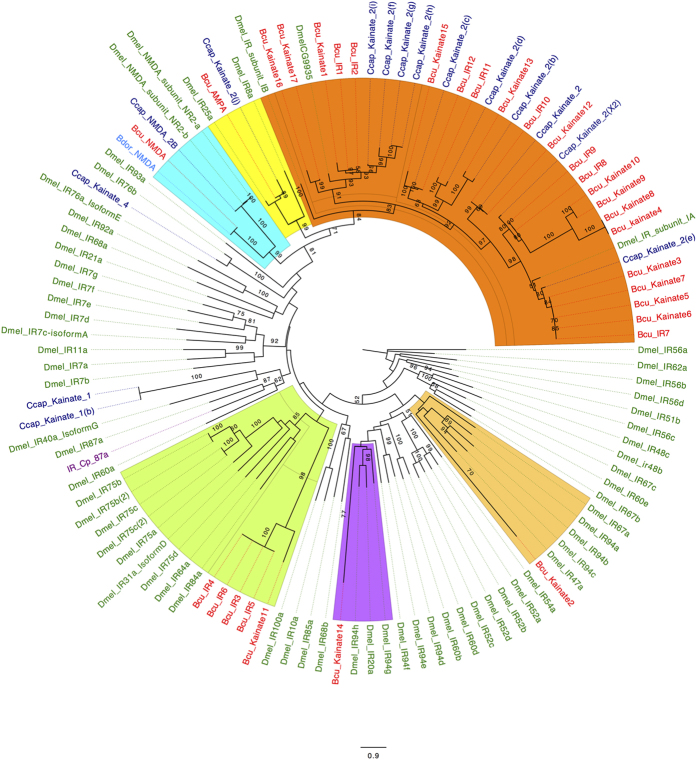
Maximum Likelihood Phylogenetic tree of Tephritid fruit flies Ionotropic Receptors Bootstrap values greater than 50% are displayed (1000 replications). (Ionotropic receptors of B. *cucurbitae*, C. *capitata*; *D. melanogaster* was used as an outgroup).

A total number of 31 putative members of the iGluR gene family were also identified from this analysis of the melon fly transcriptome (Supplementary Table S2). Based on similarities to sequences from *D. melanogaster*, we found one protein from the melon fly corresponding to the α-amino-3-hydroxy-5-methyl-4-isoxazolepropionic acid (AMPA) subfamily and one corresponding to the NMDA subfamily^23^. The remaining transcripts were classified as representing members of the Kainate subfamily. Searches against the NCBI non redundant protein (nr) database using BLASTP (protein- protein BLAST), returned hits corresponding to putatively homologous genes in *B. dorsalis* and *C. capitata*^19^,^20^ (Supplementary Table S3). These were added to our dataset and used to build a Maximum Likelihood phylogenetic tree (Fig. 5). The tree reveals three major clades: the first represents the N-Methyl-D-Aspartate (NMDA) iGluR sub-family and includes receptor sequences from *B. cucurbitae*, *B. dorsalis*. *C. capitata* and *D. melanogaster*. The second clade includes ionotropic co-receptor sequences. The third, and largest, clade has the members of the iGluR from the Kainate subfamily.

### Gustatory receptor proteins

**Figure 6:**
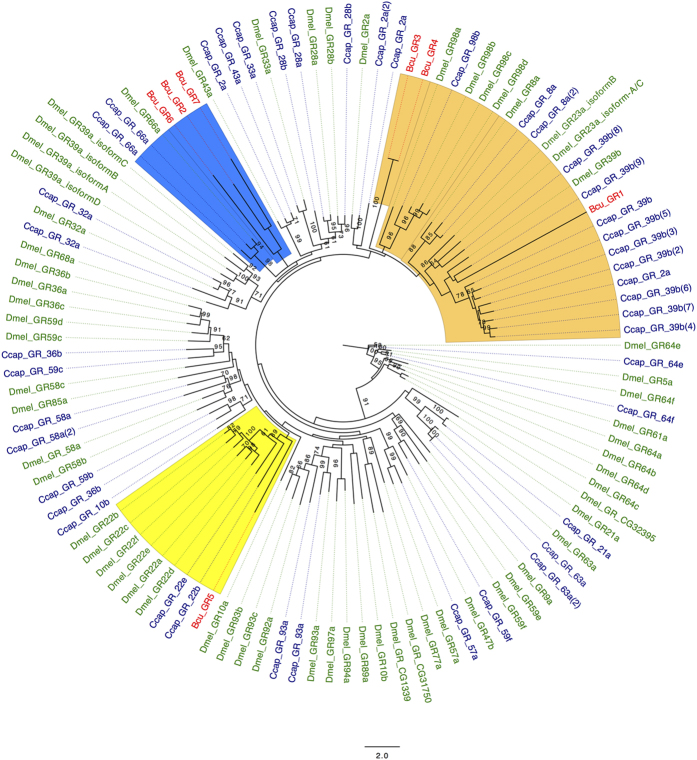
Maximum Likelihood Phylogenetic tree of Tephritid fruit flies gustatory receptors. Bootstrap values greater than 50% are displayed (1000 replications). (Gustatory receptors of: B. *cucurbitae* (BcuGR), C. *capitata* (CcapGR), D. *melanogaster* (DmelGR) was used as an outgroup).

We also identified six candidate gustatory receptor genes in the melon fly transcriptome dataset. Similar to earlier descriptions, BLAST searches of the nr sequences database returned putative homologous sequences from *C. capitata* and *D. melanogaster* (Supplementary Table S4). Figure 6 shows the Maximum Likelihood phylogenetic tree for these gustatory receptor proteins. It reveals that the gustatory receptor sequences from *B. cucurbitae* (Bcu_GR) are distributed among three different clades. The receptors Bcu_GR3 and Bcu_GR4 are present in the clade of taste sensing receptors, which includes Dmel_GR_98.

The BcuGR1 sequence clusters with the Ccap_GR_39b and DmelGR_39b sequences, which are receptors involved in mediating the acceptance or avoidance of specific substances. Finally, the Bcu_GR5 sequence is found in the clade containing theDmel_GR_22 gustatory receptors (Dmel_GR_22a, Dmel_GR_22b, Dmel_GR_22c, Dmel_GR_22d, Dmel_ GR_22e, Dmel_GR_22f). The three other melon fly gustatory receptors (Bcu_GR2, Bcu_GR6 and Bcu_GR7) cluster with Dmel_GR_66a and Ccap_GR_66a.

## Discussion

Transcriptome analysis, along with genome annotation and next-generation sequencing methods, permit the discovery and characterization of multiple genes in insects representing a wide range of functional categories^24^,^25^,^26^. Starting from the model organism *D. melanogaster*^27^ and extending to other insect species such as beetle^28^ and mosquito species^29^ among others, this approach has also been used to identify a wide range of genes involved in the specific area of chemosensory perception. Recently, this approach has also been used to identify and characterize genes in some species of the Tephritidae or true fruit flies that are major agricultural pests, including the Mediterranean fruit fly (*C. capitata*)^20^,^30^ and the oriental fruit fly (*B. dorsalis*)^19^,^31^.

Using bioinformatic tools for the analysis of transcriptome level data, and taking advantage of the existing databases^6^,^19^,^20^, we identified and characterized here a wide range of putative proteins involved in chemosensory perception in the melon fly, *B. cucurbitae.*

This species is a major pest of many cucurbit crops. Despite being a member of the genus *Bactrocera*, it infests a distinctly different set of host plants compared to the oriental fruit fly *B. dorsalis*, and has also long been known to exhibit a number of other biological differences^11^. For instance, extensive quantifications of the differential responses of these tephritid fruit flies were tested for a wide range of chemically defined kairomones such as Methyl Eugenol, a sex attractant molecule, occurring naturally in several tropical plants and widely used in male annihilation control strategies^32^. In many cases, these species showed response values that differed by up to 1,000 times or more^33^.

Our goal here was to use RNAseq to identify putative protein sequences related to olfaction and chemosensory perception in the melon fly and compare them to closely related species from the Tephritidae family.

The BLAST analysis of the OBP proteins identified in the melon fly allowed first for characterization of sequences based on the presence of specific structural features in counterparts previously described in other tephritid species along with *D. melanogaster*. Overall, when the sequences recovered here from the melon fly were compared to those of the oriental fruit fly, which is the most closely related species considered here, the percentage of identity values varied from 24 to 93%. This is consistent with levels of divergence of OBP proteins seen among other Tephritid species^19^.

Regarding their classification, the odorant binding proteins identified in the melon fly fell into either the “Classic” or the “Minus-C” sub-family. For instance, we identified a classic OBP protein in the melon fly (BcuOBP9), very similar to the OBP protein in the oriental fruit fly *B. dorsalis* (BdorOBP7), which is highly expressed in the taste sensilla on the leg, and may have a role in perceiving non-volatile chemical compounds^34^. We did not, however, identify any OBPs from the Plus-C or the dimer subfamilies in the melon fly transcriptome data. The presence of these OBPs was reported previously in the case of the oriental fruit fly *B. dorsalis*^15^ and the Mediterranean fruit fly *C. capitata*^20^,^35^,^36^.

The absence of this category in the melon fly transcriptome reflects either (1) key biological differences between these species or (2) the necessity of generating a more informative dataset, from the antennal part instead of the whole body of the insect^37^.

Within the Odorant receptors family, the *BcuOrco* putative protein showed high levels of similarity with counterparts in closely related species from the tephritidae family, which is consistent with the fact that this gene is well conserved specifically among tephritids^38^ and among insects in general^39^,^40^. The high conservation of this gene is a strong indication of its crucial role in odor detection^41^.

For the sequences classified by the GO analysis as representing ionotropic receptors (IRs), in *Drosophila* the IRs have been shown to play an important role in the detection of biological decomposed material (acids, ammonia etc.)^42^. For the melon fly, the number of IR sequences we identified is higher than those previously reported for other species^43^,^44^. This larger number may reflect the ecological specificities of the melon fly species, which feeds only on decaying or damaged fruits^45^.

The phylogenetic analysis of the Ionotropic Glutamate Receptors (iGluRs) of the melon fly and those from *D. melanogaster*^46^ along with two other tephritid species (*C. capitata* and *R. pomonella*), revealed three iGluR subfamilies. Specifically, the NMDA receptor, the Kainate receptors and the AMPA receptors, appear to be highly conserved among species that are both closely and distantly related to the melon fly.

Moreover, gustatory receptor sequences were identified in the melon fly using homology to known sequences from *D. melanogaster*, *C. capitata* and *B. dorsalis*. The melon fly sequences clustered with their *D. melanogaster* counterparts, including those previously reported to have a key role in mediating avoidance or acceptance of substrates and compounds^47^.

In addition to taste sensing, in *Drosophila,* some of these receptors also have sensory functions in organs like the abdominal ganglia^48^. To which extent these genes exhibit a similar spatial pattern of expression in the melon fly is yet to be determined. However, it is clear that four of the gustatory receptors identified from the melon fly dataset cluster with DmelGR_22a and DmelGR_66a, which are gustatory receptors specific to bitter tasting substrates in *D. melanogaster*^49^. This might have important implications in pest management efforts to control the melon fly^50^,^51^, since some bait sprays use plant-derived semiochemicals such as cucurbitacin, a toxin found in cucurbit plants. The melon fly may able to recognize these toxic compounds through its gustatory receptors.

Overall, the work presented here brings a significant contribution to the study of chemosensory receptors in tephritid fruit flies. Using a transcriptome-based approach, we were able to identify and partially characterize several important genes. These include several genes that likely play a key role in the chemosensory perception activities of the melon fruit fly. The identification and characterization of these candidate chemosensory proteins in the melon fly could be of great help in the development of novel and species specific semiochemicals used in pest management strategies^52^.

## Methods

### Insect materials, RNA purification and cDNA synthesis

The melon fly *B. cucurbitae* specimens used in this study, were from samples collected in Taiwan and were reared in the lab for several generations since 1997. Four-day melon fly adults were used for total RNA extraction with TRIzol reagent (Invitrogen, Carlsbad, CA, USA).

The purified RNA was quantified using a Nanodrop ND-2000 (Thermo Scientific, Waltham, MA, USA) and the average concentration was approximately 1.98 ng/μl (OD_260/280_ = 2.06).

### Sequencing, assembly and annotation

The RNA samples were sequenced at the Hawaii Institute for Marine Biology (HIMB) genomics core using the Illumina Genome Analyzer IIx platform. The reads were assembled using the Trinity platform and the contigs were annotated using Trinotate: an automatic functional annotation pipeline of *de novo* assembled transcriptomes (http://trinotate.sourceforge.net)^53^,^54^. Gene Ontology (GO) terms were assigned to each contig using Blast2GO^55^. The melon fruit fly transcripts were next searched for sequence homologies using BLASTX analysis in Genbank to identify candidate chemosensory protein receptors. The putative protein sequences were then compared to orthologous genes from other insect species using BLASTP^56^.

### Protein naming scheme

The putative proteins identified in the melon fly transcriptome, were labeled following the already established nomenclature^57^. The label consists of the abbreviation of the species Latin name followed by the candidate protein name and a number from 1 upwards.

### Phylogenetic analysis

Protein Sequence alignments corresponding to the melon fruit fly *B. cucurbitae* and sequences related to other tephritid fruit flies, were generated using ClustalW^58^ as implemented in Geneious V. 8.0.5 (http://www.geneious.com)^59^. These alignments served as input for the program RAxML^60^ used here to construct phylogenetic trees based on a Maximum Likelihood approach with JTT substitution model and PROTGAMMA as the GAMMA model of rate heterogeneity, with 1000 bootstrap replicates. The trees were visualized and formatted in FigTree (http://tree.bio.ed.ac.uk/software/figtree/).

## Additional Information

**How to cite this article**: Elfekih, S. *et al.* Identification and preliminary characterization of chemosensory perception-associated proteins in the melon fly *Bactrocera cucurbitae* using RNA-seq. *Sci. Rep.* **6**, 19112; doi: 10.1038/srep19112 (2016).

## Supplementary Material

Supplementary Information

## Figures and Tables

**Table 1:** Statistics of the Trinity Assembly of the Melon fruit fly transcriptome.

	*B. cucurbitae* Assembly (K25)
# of reads	44,400,736*2
Min length	201
Max length	27,802
2^nd^ long contig	27,771
3^rd^ long contig	27,766
Total length	81,038,823
Avg. length	1,469
N50	3,117
>0 bp	55,141
(>100 bp)	55,141
(>500 bp)	30,810
(>1000 bp)	21,708

**Table 2:** Classification of odorant binding proteins in *B. cucurbitae* according to the best–hit matches to OBPs in *B. dorsalis.*

ID	OBP Class	Transcript	NCBI accession	Length (AA)	Best hit	NCBI Accession	E-value	Identity%
BcuOBP1	ABPII	m.3589	KR998336	148	BdorOBP3	AGS08185	4e-06	24
BcuOBP2	Minus-C	m.5870	KR998337	150	BdorOBP10	AGS08192	3e-102	89
BcuOBP3	Classic	m.6160	KR998338	139	BdorOBP3	AGS08185	7e-65	70
BcuOBP4	Minus-C	m.6807	KR998339	142	BdorOBP10	AGS08192	2e-44	49
BcuOBP5	ABPII	m.7193	KR998340	166	BdorOBP4	AGS08186	5e-13	23
BcuOBP6	Classic	m.7840	KR998341	179	BdorOBP4	AGS08186	0.003	23
BcuOBP7	Classic	m.11279	KR998342	144	BdorOBP9	AGS08191	2e-41	45
BcuOBP8	Classic	m.11490	KR998343	155	BdorOBP9	AGS08191	1e-41	42
BcuOBP9	Classic	m.11812	KR998344	143	BdorOBP7	AGS08189	6e-104	93
BcuOBP10	Minus-C	m.16988	KR998345	160	BdorOBP1	AGS08192	2e-14	27
BcuOBP11	ABPII	m.51638	KR998346	146	BdorOBP5	GS08187	2e-06	20
BcuOBP12	Classic	m.1076	KR998347	136	BdorOBP5	GS08187	2e-17	32
BcuOBP13	Classic	m.2892	KR998348	173	BdorOBP5	GS08187	2e-11	28
