# Supplementary material for: Identification and preliminary characterization of chemosensory perception-associated proteins in the melon fly Bactrocera cucurbitae using RNA-seq
Source: Sci Rep. 2016 Jan 11;6:19112. doi: 10.1038/srep19112 (PMC4707516; doi:10.1038/srep19112)
Supplement: Supplementary Information [file srep19112-s1.pdf]

Identification and preliminary characterization of chemosensory perception-associated proteins in the melon fly *Bactrocera cucurbitae* using RNA-seq

Samia Elfekih[1*], Chien-Yu Chen[2], JuChun Hsu[3], Mahdi Belcaid[4] & David Haymer[5]

[1] Commonwealth Science and Industry Organization (CSIRO), Biosecurity flagship P.O. BOX 1700 Canberra ACT 2601, Australia,

[2] National Taiwan University, Department of Bio-industrial Mechatronics and Engineering, Taipei, Taiwan,

[3] National Taiwan University, Department of Entomology, National Taiwan University Taipei, Taiwan,

[4]Information and Computer Sciences, University of Hawaii at Manoa, Honolulu, Hawaii, USA,

[5]Department of Cell and Molecular Biology, University of Hawaii at Manoa, Honolulu, Hawaii, USA

[*]Corresponding author: S.E. (samia.elfekih@csiro.au)

(a)

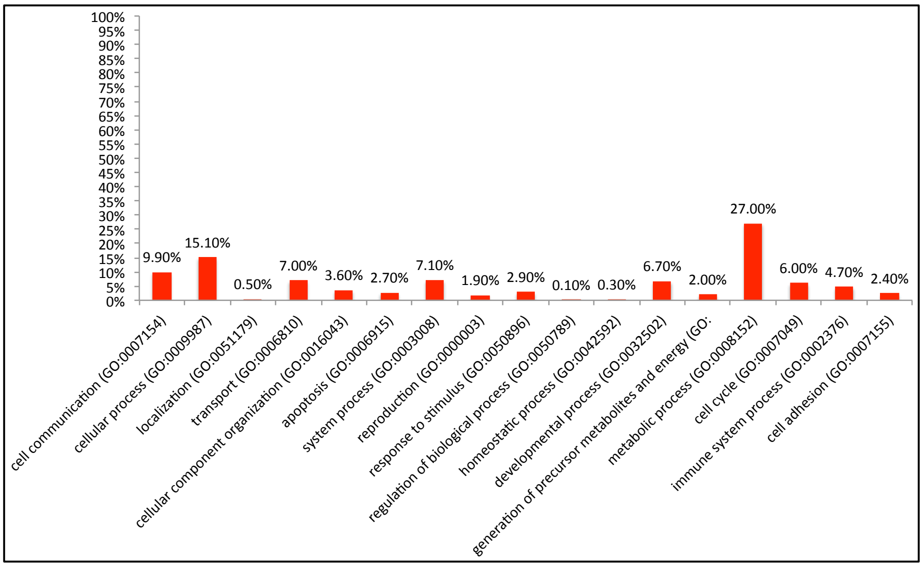

(b)

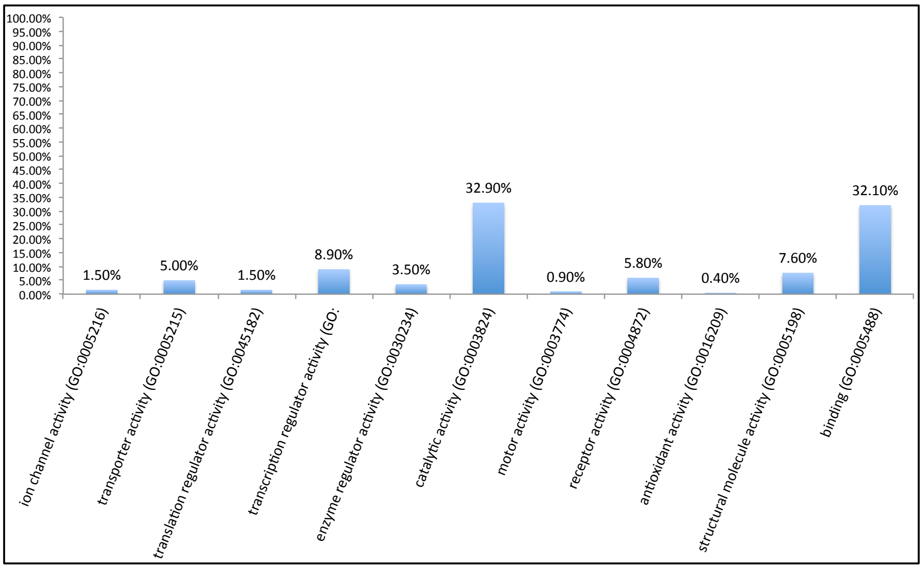

(c)

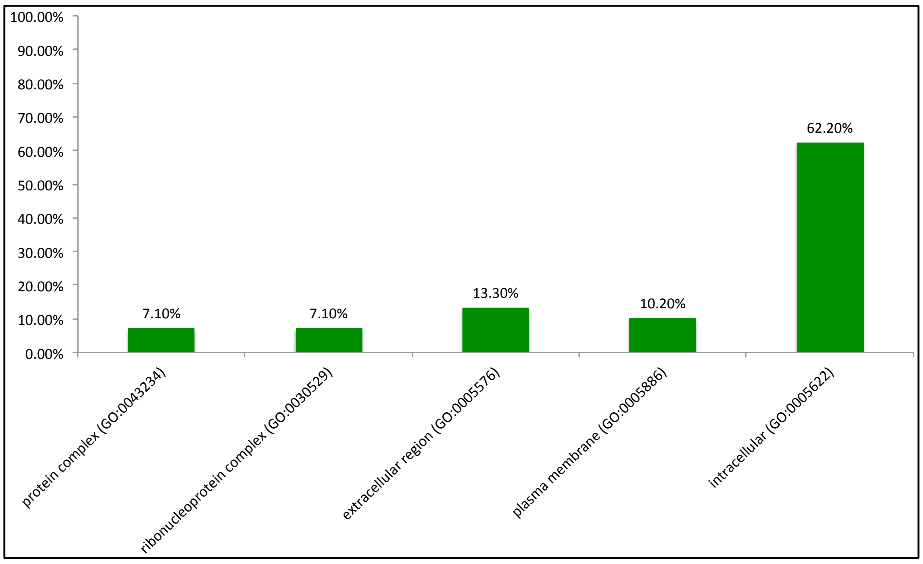

Supplementary Fig. S1. Gene Ontology (GO) results. GO analysis corresponding to 25943 sequences in *B. cucurbitae* involved in Biological process (a), Molecular function (b), and cellular component (c). The y-axis in each of the three graphs corresponds to the percentage of transcripts in each GO category.

Supplementary table S1. Genbank accession codes corresponding to the representative sequences of Odorant-Binding Proteins in other fruit fly species used for comparison purposes.

Species	ID	Genbank Accession
Drosophila melanogaster	Dmel_OBP83cd	NP_649612
	Dmel_OBP83ef	NP_731042
	Dmel_OBP_56c	NM_166374.4
	Dmel_OBP_47a	NM_206088.1
	Dmel_OBP_56f	NM_166375.3
	Dmel_OBP_85a	NM_141545.2
	Dmel_OBP_56i	NM_001202051.1
	Dmel_OBP_84a	NM_001104230.3
	Dmel_OBP_51a	NM_166079.2
	Dmel_OBP_19b	KP743683.1
	Dmel_OBP_57c	KP743697.1
	Dmel_OBP_56g	NM_206181.3
	Dmel_OBP_22a	NM_164447.3
	Dmel_OBP_73a	NM_001104158.1
	Dmel_OBP_56a	KP743692.1
	Dmel_OBP_56d	NM_001299690.1
	Dmel_OBP_56e	NM_001299691.1
	Dmel_OBP_59a	NM_176249.3
	Dmel_OBP_58d	NM_137867.2
	Dmel_OBP_50b	NM_166051.3
	Dmel_OBP_93a	KP236520.1
	Dmel_OBP_46a	NM_136730.1
	Dmel_OBP_47b	NM_136825.2
	Dmel_OBP_50a	NM_206110.2
	Dmel_OBP_50d	NM_166053.3
	Dmel_OBP_49a	NM_136968.2
	Dmel_OBP_50e	NM_137115.3
	Dmel_OBP_58b	NM_137865.3
	Dmel_OBP_58c	NM_137866.2
	Dmel_OBP_56h	NM_001202050.1
	Dmel_OBP_50c	NM_166052.3
	Dmel_OBP_69a	NM_079315.2
	Dmel_OBP_83a	NM_001300261.1
	Dmel_OBP_83b	NM_079518.3
	Dmel_OBP_76a	NM_079438.3
	Dmel_OBP_19a	NM_167700.3
	Dmel_OBP_56b	NM_137599.2
	Dmel_OBP_28a	NM_078781.3
	Dmel_OBP_19c	NM_167701.2
	Dmel_OBP_18a	NM_133122.2
	Dmel_OBP_57e	NP_611488.1
	Dmel_OBP_57d	NP_725973.1
	Dmel_OBP_57b	NP_001286632.1
	Dmel_OBP_57a	NP_725966.1
	Dmel_OBP_99d	NP_001263078.1
Ceratitis capitata	Ccap_0BP_99c	XP_004521186.1
	Ccap_0BP_99d	XM_004521127.1
	Ccap_0BP_8a	XM_004521128.1
	Ccap_0BP_56d	XM_004517746.1
	Ccap_0BP_49a	XM_004522926.1
	Ccap_0BP_69a	NP_001295335.1
	Ccap_0BP_83a-1	NP_001295333.1
	Ccap_0BP_83a-2	CDJ79887.1

	Ccap_0BP_19b<br>Ccap_0BP_19d-1<br>Ccap_0BP_19d-2<br>Ccap_0BP_28a<br>Ccap_0BP_56h<br>Ccap_0BP_19a<br>Ccap_0BP_84a-1<br>Ccap_0BP_44a	XM_004524970.1<br>XM_004524978.1<br>XM_004525083.1<br>XM_004524959.1<br>XP_004517804.1<br>XM_004524969.1<br>XM_004529312.1<br>XM_004535885.1
Rhagoletis pomonella	Rp_OBP_56h<br>Rp_OBP_50e<br>Rp_OBP_56a<br>Rp_OBP_99c<br>Rp_OBP_49a<br>Rp_OBP_19b<br>Rp_OBP_83g<br>Rp_OBP_44a<br>Rp_OBP_56d<br>Rp_OBP_99b	EZ138985.1<br>EZ131620<br>EZ139735<br>EZ138892.1<br>EZ118317<br>EZ138033.1<br>EZ136242<br>EZ137697.1<br>EZ116239<br>EZ135703.1
Rhagoletis suavis	Rs_OBP_5<br>Rs_OBP_1<br>Rs_OBP_3<br>Rs_OBP_2<br>Rs_OBP_7<br>Rs_OBP_8<br>Rs_OBP_9<br>Rs_OBP_4<br>Rs_OBP_6	EX453824<br>EX453819<br>EX453821<br>EX453820.1<br>EX453827<br>EX453830<br>EX453831<br>EX453822<br>EX453825
Bactrocera dorsalis	BdorOBP3<br>BdorOBP10<br>BdorOBP3<br>BdorOBP10<br>BdorOBP4<br>BdorOBP4<br>BdorOBP9<br>BdorOBP9<br>BdorOBP7<br>BdorOBP1<br>BdorOBP5<br>BdorOBP5<br>BdorOBP5	AGS08185<br>AGS08192<br>AGS08185<br>AGS08192<br>AGS08186<br>AGS08186<br>AGS08191<br>AGS08191<br>AGS08189<br>AGS08192<br>GS08187<br>GS08187<br>GS08187

Supplementary Table S2. Genbank accession codes corresponding to the representative sequences of Ionotropic and Gustatory receptors in the melon fly *B. cucurbitae.*

	ID	Genbank Accession
Gustatory receptors	Bcu_GR1	KR998350
	Bcu_GR2	KR998351
	Bcu_GR3	KR998352
	Bcu_GR4	KR998353
	Bcu_GR5	KR998354
	Bcu_GR6	KR998355
Ionotropic receptors	Bcu_AMPA	KR998356
	Bcu_NMDA	KR998357
	Bcu_IR1	KR998358
	Bcu_IR2	KR998359
	Bcu_IR3	KR998360
	Bcu_IR4	KR998361
	Bcu_IR5	KR998362
	Bcu_IR6	KR998363
	Bcu_IR7	KR998364
	Bcu_IR8	KR998365
	Bcu_Kainate_1	KR998366
	Bcu_Kainate_2	KR998367
	Bcu_Kainate_3	KR998368
	Bcu_Kainate_4	KR998369
	Bcu_Kainate_5	KR998370
	Bcu_Kainate_6	KR998371
	Bcu_Kainate_7	KR998372
	Bcu_Kainate_8	KR998373
	Bcu_Kainate_9	KR998374
	Bcu_Kainate_10	KR998375
	Bcu_Kainate_11	KR998376
	Bcu_Kainate_12	KR998377
	Bcu_Kainate_13	KR998378
	Bcu_Kainate_14	KR998379

Supplementary table S3. Genbank accession codes corresponding to the representative sequences of Ionotropic receptors in other fruit fly species used for comparison purposes.

	ID	Genbank Accession
Drosophila melanogaster	Dmel_IR_100a	AAF57202.2
	Dmel_IR_10a	ABW09389.1
	Dmel_IR_11a	AAF48158.2
	Dmel_IR_20a	AAF50808.1
	Dmel_IR_21a	AAF51569.2
	Dmel_IR_25a	ADU79032.1
	Dmel_IR_31a_isoformD	AAN10751.2
	Dmel_IR_40a_isoformG	AHN54646.1
	Dmel_IR_47a	AAF58803.1
	Dmel_IR_48b	AAF58625.2
	Dmel_IR_48c	AAM68696.1
	Dmel_IR_51b	AAM68524.1
	Dmel_IR_52a	AAF58132.2
	Dmel_IR_52b	AAM70972.2
	Dmel_IR_52c	AAM70973.1
	Dmel_IR_52d	AAF58131.3
	Dmel_IR_54a	AAF57800.2
	Dmel_IR_56a	AAM68433.1
	Dmel_IR_56b	AAF57539.1
	Dmel_IR_56c	AAF57538.1
	Dmel_IR_56d	AAF57537.1
	Dmel_IR_60a	AAF47186.1
	Dmel_IR_60b	ACL83208.1
	Dmel_IR_60d	ACL83210.2
	Dmel_IR_60e	AAF47222.3
	Dmel_IR_62a	ABC66126.1
	Dmel_IR_64a	AAF50781.1
	Dmel_IR_67a	AAF50247.3
	Dmel_IR_67b	AAF50158.1
	Dmel_IR_67c	NP_729609.1
	Dmel_IR_68a	NP_001287031.1
	Dmel_IR_68b	NP_648548.1
	Dmel_IR_75a	NP_649012.2
	Dmel_IR_75b(2)	NP_001137966.2
	Dmel_IR_75c	NP_649013.3
	Dmel_IR_75d	NP_649074.2
	Dmel_IR_76a_IsoformE	NP_001097647.3
	Dmel_IR_76b	NP_649176.1
	Dmel_IR_7a	NP_572406.1
	Dmel_IR_7b	NP_572410.2
	Dmel_IR_7c-isoformA	NP_572411.1
	Dmel_IR_7d	NP_001138175.1
	Dmel_IR_7e	NP_001138176.1
	Dmel_IR_7f	NP_001138177.1
	Dmel_IR_7g	NP_572413.2
	Dmel_IR_84a	NP_649720.2
	Dmel_IR_85a	NP_649833.1
	Dmel_IR_87a	NP_650290.2
	Dmel_IR_8a	NP_727328.1
	Dmel_IR_92a	NP_001097845.2
	Dmel_IR_93a	NP_650924.3
	Dmel_IR_94a	NP_732699.1
	Dmel_IR_94b	NP_732700.2

	Dmel_IR_94c	NP_732701.2
	Dmel_IR_94d	NP_001138099.1
	Dmel_IR_94e	NP_001097885.2
	Dmel_IR_94f	NP_732868.2
	Dmel_IR_94g	NP_651147.2
	Dmel_IR_94h	NP_651148.2
	Dmel_IR_subunit_IA	gb|AAF50652.2
	Dmel_IR_subunit_IB	gb|AAF50306.2
	Dmel_NMDA_subunit_NR2_a	gb|AAL12478.1
	Dmel_NMDA_subunit_NR2_b	gb|AAL12479.1
	DmelCG9935	NP_001036251.2
	Ccap_kainate_1(b)	gb|JAC03796.1
	Ccap_kainate_1	XP_004520203.1
	Ccap_NMDA_2B	XP_004521039.1
	Ccap_kainate_2(f)	XP_004518939.1
	Ccap_kainate_2(g)	gb|JAC03798.1
	Ccap_kainate_2(h)	gb|JAC03797.1
	Ccap_kainate_2(i)	gb|JAC03795.1
	Ccap_kainate_2(X2)	XP_004523567.1
	Ccap_kainate_2(e)	XP_004523576.1
	Ccap_kainate_2	XP_004531081.1
	Ccap_kainate_2(d)	XP_004524631.1
	Ccap_kainate_2(b)	XP_004537774.1
	Ccap_kainate_2(c)	XP_004531079.1
	Ccap_kainate_2(j)	gb|JAC01321.1

Supplementary table S4. Genbank accession codes corresponding to the representative sequences of gustatory receptors in other fruit fly species used for comparison purposes.

	ID	Genbank Accession
Drosophila melanogaster	Dmel_GR39b	P58960.1
	Dmel_GR23a_isoformB	P83292.1
	Dmel_GR23a_isoformA/C	Q9VQE7.3
	Dmel_GR8a	NP_511097.3
	Dmel_GR98a	NP_651564.1
	Dmel_GR98b	NP_733213.1
	Dmel_GR98c	NP_524531.2
	Dmel_GR98d	NP_733214.1
	Dmel_GR2a	Q9W594.3
	Dmel_GR68a	NP_524027.2
	Dmel_GR32a	NP_523543.3
	Dmel_GR39a_isoformD	AAN11116.1
	Dmel_GR39a_isoformA	NP_724332.1
	Dmel_GR39a_isoformB	AAN11114.1
	Dmel_GR39a_isoformC	AAN11115.1
	Dmel_GR66a	P_523971.3
	Dmel_GR33a	NP_525102.4
	Dmel_GR43a	NP_523650.2
	Dmel_GR28a	NP_523504.2
	Dmel_GR28b	Q9VM08.2
	Dmel_GR9a	NP_727392.1
	Dmel_GR_CG323995	CG323995.1
	Dmel_GR21a	ABK97615.1
	Dmel_GR63a	NP_001137883.1
	Dmel_GR64b	NP_728921.1
	Dmel_GR64d	Q9VZJ6.2
	Dmel_GR64c	CBA14206.1
	Dmel_GR61a	CBA14259.1
	Dmel_GR64a	NP_728920.1
	Dmel_GR64e	P83296.2
	Dmel_GR64f	NP_728924.2
	Dmel_GR5a	NP_511050.1
	Dmel_GR47b	NP_725040.2
	Dmel_GR57a	NP_523798.1
	Dmel_GR77a	NP_730560.1
	Dmel_GR_CG31750	Q8INZ1.1
	Dmel_GR_CG1339	NP_610307.1
	Dmel_GR10b	9VYZ2.1
	Dmel_GR89a	Q9VEU0.2
	Dmel_GR94a	Q8IMZ5.1
	Dmel_GR97a	Q8IMQ6.1
	Dmel_GR93a	AAF55923.2
	Dmel_GR92a	Q8IN58.2
	Dmel_GR93c	Q9VD74.2
	Dmel_GR93b	Q8IN23.2
	Dmel_GR10a	NP_727523.1
	Dmel_GR22d	P84181.1
	Dmel_GR22a	P58951.2
	Dmel_GR22e	NP_722731.1
	Dmel_GR22f	P58954.1
	Dmel_GR22c	P58952.3
	Dmel_GR22b	P84180.1
	Dmel_GR58b	Q9W2B1.2
	Dmel_GR58a	P58962.1

	Dmel_GR85a	Q8INM9.1
	Dmel_GR58c	Q9W2B2.3
	Dmel_GR59c	Q9W1U5.2
	Dmel_GR59d	P58985.1
	Dmel_GR36c	Q8INZ2.1
	Dmel_GR36a	P58955.1
	Dmel_GR36b	Q9VJF2.2
Ceratitis capitata	Ccap_GR_39b(9)	XP_004534028.1
	Ccap_GR_39b	XP_004536483.1
	Ccap_GR_39b(5)	XP_004536478.1
	Ccap_GR_39b(3)	XP_004536480.1
	Ccap_GR_39b(2)	XP_004536482.1
	Ccap_GR_2a	XP_004519274.1
	Ccap_GR_2a*	XP_004536481.1
	Ccap_GR_2a**	XP_004536475.1
	Ccap_GR_39b(6)	XP_004536477.1
	Ccap_GR_39b(7)	XP_004536476.1
	Ccap_GR_39b(4)	XP_004536479.1
	Ccap_GR_39b(8)	XP_004536474.1
	Ccap_GR_8a	XP_004536506.1
	Ccap_GR_8a(2)	XP_004536505.1
	Ccap_GR_98b	XP_004533711.1
	Ccap_GR_32a	XP_004533391.1
	Ccap_GR_32a*	XP_004526692.1
	Ccap_GR_66a	XP_004525752.1
	Ccap_GR_66a*	XP_004529782.1
	Ccap_GR_28b	XP_004531567.1
	Ccap_GR_59f	XP_004526066.1
	Ccap_GR_28b*	XP_004531432.1
	Ccap_GR_21a	XP_004517826.1
	Ccap_GR_63a	XP_004524608.1
	Ccap_GR_64e	XP_004531638.1
	Ccap_GR_64f	XP_004531639.1
	Ccap_GR_57a	XP_004526569.1
	Ccap_GR_93a	XP_004530764.1
	Ccap_GR_22b	XP_004521913.1
	Ccap_GR_22e	XP_004521918.1
	Ccap_GR_10b	XP_004534880.1
	Ccap_GR_36b	XP_004534878.1
	Ccap_GR_59b	XP_004534879.1
	Ccap_GR_58a(2)	XP_004520352.1
	Ccap_GR_58a	XP_004520353.1
	Ccap_GR_59c	XP_004534883.1
	Ccap_GR_36b	XP_004534882.1
